# New Appliances and Things Medical

**Published:** 1911-03-04

**Authors:** 


### NEW APPLIANCES & THINGS MEDICAL.

### THE MAGIC DOOR OPENER.

(Domestic Appliances Ltd., 30 Winchmore Street, Cheltenham.)

This ingenious bit of apparatus, which can easily be fixed to any door, is a simple mechanism which is operated by the foot. A person having his hands full—as a nurse bearing a tray, or a dresser with instruments in his hands—can open a closed door to which the appliance is fixed quite easily without having to stop and turn the door handle. The door opener is supplied in two sets, one for fixing to the inside of the door so as to enable the latter to be opened from within, the other for use from the exterior. The fittings are of a superficial nature, are not connected with the floor, do not interfere with the door in any way, and do not detract from the appearance of the door either when closed or shut. For institutional use the "Magic Door Opener" seems admirable. It is when one has grasped its simplicity and easy method of working that one wonders how it is that no one has thought of applying the ingenious principle on which it is founded before. Full diagrams and explanatory literature of this new patent may be obtained on application to the makers.

### PROTOCAS.

(Lewis and Burrows, Ltd., Scientific Chemists, London.)

Protocas is a combination of milk proteids with glycerophosphates, which is recommended as a food in cases of debility and neurasthenia. It is in the form of a white powder, free from purin, starch, sugar, and fat, and consists largely of pure milk proteid which has been enriched by the addition of glycerophosphates. The dose is two teaspoonfuls, beaten up with a little cold water and added to any non-acid food or drink. Prepared in this way it is free from any unpleasant flavour, and it is undoubtedly a highly sustaining, nourishing, and stimulating food. In cases of diabetes associated with wasting, and in cases of marasmus it is well worth a trial, while it is a useful food stimulant to brain workers. Samples may be obtained on application to the makers at the head office, 146 Holborn Bars, E.C. The price varies from 11d. to 5s. 6d. per tin.

